# Isolation of Endogenously Assembled RNA-Protein Complexes Using Affinity Purification Based on Streptavidin Aptamer S1

**DOI:** 10.3390/ijms160922456

**Published:** 2015-09-16

**Authors:** Yangchao Dong, Jing Yang, Wei Ye, Yuan Wang, Chuantao Ye, Daihui Weng, Huan Gao, Fanglin Zhang, Zhikai Xu, Yingfeng Lei

**Affiliations:** Department of Microbiology, Faculty of Preclinical Medicine, the Fourth Military Medical University, Xi’an 710032, China; E-Mails: ycdongfmmu@gmail.com (Y.D.); cherry.yj@gmail.com (J.Y.); ahjxyw@163.com (W.Y.); judy_625@163.com (Y.W.); yechuantao2008@163.com (C.Y.); paradise_009@163.com (D.W.); gaohuan954227274@163.com (H.G.); flzhang@fmmu.edu.cn (F.Z.)

**Keywords:** living mammalian cells, DENV, streptavidin aptamer S1, affinity purification, RNA binding protein, 40S ribosomal protein S8

## Abstract

Efficient isolation of endogenously assembled viral RNA-protein complexes is essential for understanding virus replication mechanisms. We have developed an affinity purification strategy based on an RNA affinity tag that allows large-scale preparation of native viral RNA-binding proteins (RBPs). The streptavidin-binding aptamer S1 sequence was inserted into the 3′ end of dengue virus (DENV) 5′–3′ UTR RNA, and the DENV RNA UTR fused to the S1 RNA aptamer was expressed in living mammalian cells. This allowed endogenous viral ribonucleoprotein (RNP) assembly and isolation of RNPs from whole cell extract, through binding the S1 aptamer to streptavidin magnetic beads. Several novel host DENV RBPs were subsequently identified by liquid chromatography with tandem mass spectrometry (LC-MS/MS), including RPS8, which we further implicate in DENV replication. We proposed efficient S1 aptamer-based isolation of viral assembled RNPs from living mammalian cells will be generally applicable to the purification of high- and low-affinity RBPs and RNPs under endogenous conditions.

## 1. Introduction

It was well known that host RNA-binding proteins (RBPs) are involved in the viral replication for plus-strand (+) RNA viruses. A limited number of host RBPs affect viral RNA recruitment for replication, help assemble the replicase complexes, control viral RNA synthesis, or affect viral RNA stability [1]. Interestingly, the untranslated regions (UTRs) of virus RNA are usually highly structured and they are involved in regulation of viral RNA translation and replication [2,3]. Further identification of host factors interacting with viral RNA UTRs will greatly improve our understanding of viral biology and pathology. Discovering viral replication mechanisms in cells will require generalizable methods that isolate the RBPs or ribonucleoprotein (RNP) complexes in a pure and functionally intact form.

Identifying RBPs or RNPs could be facilitated by a highly specific RNA affinity tag that allows selective recovery of all proteins associated with the tagged RNA under nondenaturing conditions. However relatively few RNA affinity tags have been identified. Examples include the specific RNA-hairpin structure that binds to bacteriophage MS2 coat protein, nascent “boxB” hairpins from bacteriophages λ and P22, “StreptoTag” which binds to streptomycin [4,5], J6f1 RNA aptamer which binds to tobramycin [6] ENREF 6 and S1 RNA aptamer developed by Srisawat and Engelke which binds to streptavidin [7]. Biotin can be used to elute the RNA aptamer S1 from streptavidin and this system was successfully used to isolate the ribonucleoprotein enzyme RNase P from cell extract [7].

Based on well-developed RNA affinity tags, many studies utilized cell extracts and synthetic target RNA to assemble the RNA-protein complexes *in vitro* for isolation of RBPs. The “Glutathione RNA Affinity Chromatography” approach is a typical example to identify proteins specifically associated with target RNA [8,9]. Generally, the boxB hairpin sequences were inserted into the 3′ UTR of target RNAs, and the 21 amino acid λ peptide was fused with glutathione *S*-transferase (GST). By binding the GST-λ peptide fusion protein to the hybrid RNA including boxB hairpin RNA and then incubating with cell extracts, RNA-protein complexes were assembled on boxB-containing RNAs. Recently, Ward *et al.* [10] reported a new strategy to identify DENV 5′ UTR and 3′ UTR RBPs. In this method, the tobramycin RNA aptamer was incorporated into DENV 5′ and 3′ UTRs containing RNA and then bound to a tobramycin matrix reversibly, afterward the RNA-tobramycin matrix was incubated with SILAC-labeled cell lysates, and bound proteins are eluted. However, due to the possible incorrect fold of synthetic RNA and nonspecific RNA-protein interactions, the *in vitro*-assembled RNA-protein complexes may not represent authentic RNPs which existed under physiological conditions.

To avoid potential biochemical artifacts and circumvent limitations of the *in vitro* approach, several new methods reportedly have been used to identify authentic RNA-interacting proteins from living cells [11,12,13,14,15,16,17]. Francois *et al.* [17] introduced the S1 aptamer into snR30 snoRNA and isolated the endogenously-assembled snR30 snoRNP from yeast cells. Hogg *et al.* [14] developed an efficient and generalizable method for affinity purification of RNPs assembled in living mammal cells for the first time. The 7SK RNA tagged by RNA Affinity in Tandem (RAT), containing PP7 and Tob sequences, was expressed in cells, allowing endogenous assembly of the tagged RNA into RNPs and purifying tagged RNPs from whole cell extract. Lee *et al.* [18] created the mutated Csy4 endoribonuclease, which specifically binds irreversibly to a 16-nt hairpin sequence of PP7 RNA, and this interaction is readily reversible in the presence of imidazole. After the biotinylated Csy4 H29A/S50C was immobilized on Avidin agarose, the protein partners specifically binding to Csy4 hairpin-tagged RNA were selectively purified. This RNA affinity purification strategy offers an easy elution procedure and is simple and effective for transcriptome analysis.

Formaldehyde and 254 nm UV light cross-linking are commonly used to identify RNA-protein interactions in previously reported strategies [19]. To determine the proteins that directly bind to mRNAs in living Hela cells, Castello *et al.* [20] “froze” protein-mRNA interactions by covalent UV cross-linking. The cells were cultured in the presence of photoactivatable nucleotide 4-thiouridine (4-SU) which was thus incorporated into nascent RNAs, and efficiently crosslinked by 365 nm UV light irradiation. Alexander *et al.* [21] also applied the photoreactive nucleoside-enhanced UV crosslinking and oligo (dT) affinity purification approach to identify the sites of protein-mRNA interactions in mammalian cells. A key feature of this approach is the incorporation of photoreactive nucleoside analogs, 4-SU and 6-thioguanosine (6-SG), to metabolically label cellular RNA. This cross-link between protein and RNA is more effective than 254 nm UV light cross-linking and formaldehyde cross-linking and does not promote protein-protein cross-linking.

In this study, we present a new affinity purification variation based on using the streptavidin-binding RNA aptamer S1 as a tag to isolate DENV-2 (strain Tr1751) RNA-binding proteins in living mammalian cells as demonstration. The S1 RNA aptamer sequence was cloned into the 3′ end of DENV RNA UTR in a plasmid, and after transfection the hybrid RNA was transcribed in living cells*.* This allowed endogenous viral RNP assembly and isolation of RNPs from whole cell extract. By using this method, we identified several novel host proteins binding to DENV RNA. This method, coupled with LC-MS/MS, will allow quick and easy purification of high- and low-affinity RBPs and RNPs under endogenous conditions.

## 2. Results

### 2.1. Design and Expression of Plasmid-Based S1-Aptamer-Tagged 5′–3′ UTR RNA

**Figure 1:**
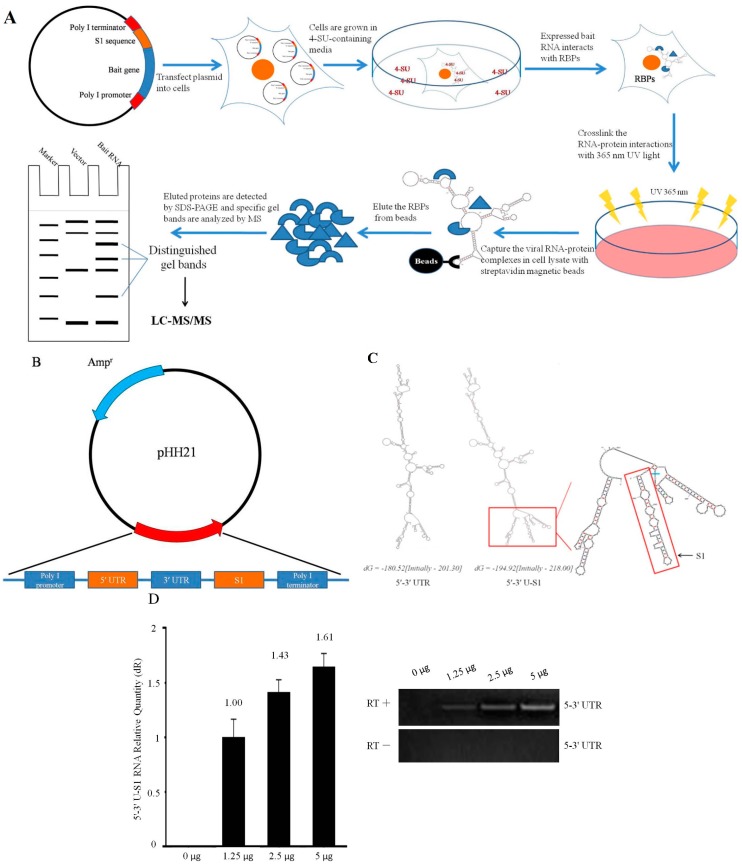
(**A**) Schematic of experimental strategy to purify S1 aptamer-tagged vRNAs and endogenously-assembled RNA-protein complexes under non-denaturing conditions; (**B**) Cloning strategy for plasmid to express aptamer-tagged viral RNAs. The 3′ end of DENV 5′–3′ UTR RNA was tagged with S1 aptamer (that binds streptavidin) and subcloned into the pHH21 vector backbone between the RNA polymerase I promoter and polymerase I terminator; (**C**) The secondary structures of DENV 5′–3′ UTR and 5′–3′ U-S1 sequence were predicted by RNAfold. Red boxes show the region where the S1 sequence is cloned in the context of the full structure (with a close up). The free energies are reported as “dG = yy.y [initially -xx.x]”, where “yy.y” is the revised free energy, and “xx.x” is the “initial dG” found by the folding algorithm (detail elucidation is available online) [22]. The S1 sequence folds into a distinct secondary structure that is unlikely to impede the natural folding of the virus; (**D**) Plasmid Dose-dependent expression of RNA. Endogenously-expressed 5′–3′ U-S1 RNAs were quantified by RT-qPCR with primers designed against the 5′–3′ UTR segment, which contains both 5′ and 3′ UTR segments, using total RNA from transiently transfected 293T cells. RNA yield depended on the amount of plasmid that is shown in the left chart. The transfection efficiency was routinely >70%. The right one shows the efficacy of DNAse digestion of plasmid DNA. RT-PCR is performed in the presence and absence transcriptase with primers designed against DENV 5′ UTR.

An overview of the experimental steps of the RNA pull-down assay devised to isolate endogenously-assembled viral RNP complexes from cells is given in Figure 1A. Generally, the S1 RNA aptamer sequence was cloned into a plasmid in the 3′ end of bait viral RNA flanked by polymerase I promoter and polymerase I terminator. The plasmid was transfected and the hybrid RNA was thus transcribed in 293T cells in the presence of the photoactivatable nucleotide 4-SU which was thus incorporated. After endogenous viral RNP was assembled, the RNA-protein complexes were cross-linked by 365 nm UV light irradiation and the RNPs were isolated from whole cell extract through binding of S1 aptamer to Streptavidin magnetic beads. The eluted proteins were then detected by SDS-PAGE and analyzed with LC-MS/MS.

To express the DENV 5′–3′ UTR RNA in 293T cells*,* the viral RNA genes were cloned into pHH21 plasmids and fused with S1 RNA affinity tags (Figure 1B). The most stable secondary structures of hybrid DENV 5′–3′ UTR and 5′–3′ U-S1 sequences were calculated using the RNAfold web server [22] with the default parameters and the result for minimum free energy prediction showed that the inserted S1 sequence likely has no significant effect on the secondary structures of 5′–3′ UTR (Figure 1C). To confirm whether the correct RNA products were transcribed when plasmid pHH21-5′–3′ U-S1 was transfected into living cells, 1.25, 2.5 and 5 μg plasmids were used per 35 mm cell culture dish, respectively. Then, the total RNA was extracted and the 5′–3′ U-S1 RNA level was quantified by RT-qPCR with primers targeting the DENV 5′–3′ UTR segment. The results showed that DENV 5′–3′ U-S1 RNA was transcribed as one intact RNA (Figure S1) and the amount of transcrption in 293T cells was dose-dependent (Figure 1D). And according to the *C*_t_ value (Table S1) in Figure 1D, the copy number of 5′–3′ U-S1 RNA transcribed in cells under polymerase I promoter is roughly equivalent to the amount of β-actin mRNA. This demonstrates that the pHH21 vector, containing the human RNA polymerase I promoter and the mouse RNA polymerase I terminator, is an effective system to generate intact viral RNA segments in living mammalian cells.

### 2.2. Isolating Endogenously-Assembled DENV 5′–3′U-S1 RNPs

**Figure 2:**
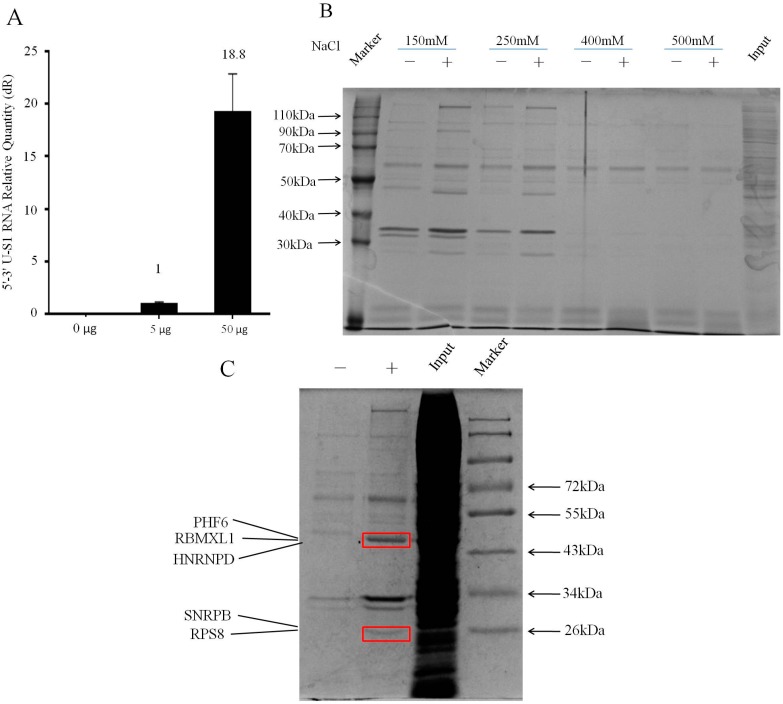
Purification of endogenously-assembled 5′–3′ U-S1 RNPs. (**A**) The 5′–3′ U-S1RNAs eluted from streptavidin beads were detected by RT-qPCR with different amount of plasmid shown. In order to normalize the RT-qPCR, an equal amount of β-actin RNA was added to both groups prior to isolating RNA from beads; (**B**) In the RNA pull down assay, the wash buffer containing different NaCl concentrations was applied to explore the perfect wash conditions for affinity purification. Proteins bound to washed streptavidin beads from 5′–3′ U-S1 RNA and mock purifications were resolved by SDS-PAGE and visualized by Coomassie blue staining. Lanes marked with a minus are untransfected controls; (**C**) The two distinguished gel bands of the 5′–3′ U-S1 RNA purification compared to mock purification were subjected to mass spectrometry, and five proteins were identified.

To investigate whether hybrid RNA with DENV 5′–3′ U and S1 RNA aptamer, assembled in living mammalian cells, was able to bind to streptavidin C1 magnetic beads, we tested the amount of 5′–3′ U RNA after affinity purification with streptavidin [7]. 5 and 50 μg plasmid pHH21-5′–3′ U-S1 was transfected into 293T cells in 150 mm dish, respectively. Twenty-four hours later, cells were collected by centrifugation followed by rapid lysis. A cleared lysate was then directly incubated with pre-treated streptavidin C1 magnetic beads. Upon binding the S1-tagged RNA species to streptavidin beads, the beads were extensively washed to remove non-specifically bound RNA. Finally, the tagged 5′–3′ U-S1 RNAs were isolated and subjected to RT-qPCR with primers directed against the DENV 5′–3′ UTR segment. As shown in Figure 2A, more hybrid DENV 5′–3′ U-S1 RNA was transcribed in 293T cells and more was captured by the beads, when more plasmid was used. This result suggests that the endogenously-generated 5′–3′ U-S1 segment could specifically bind to streptavidin C1 magnetic beads.

Next, we performed the protocol on a larger scale (about 1 × 10^8^ cells) with a vector control. Five dishes of cells were transfected with either pHH21-5′–3′ U-S1 or pHH21 for 6 h, and incubated with 4-SU containing medium for additional 18 h. After, we cross-linked viral RNA-protein complexes with 365 nm UV light irradiation. After the cells were lysed, the viral RNA-protein complexes were purified from the lysates by binding to streptavidin magnetic beads. The wash conditions of affinity purification were explored by changing the NaCl concentrations in wash buffer (Figure 2B). Two specific bands were seen in the transfected cells in buffer containing 250 mM NaCl but these were lost at higher concentration; therefore, we deemed this concentration to be the appropriate one for the RNA pull-down assay. To determine the identities of the resulting purified samples in the 5′–3′ U-S1 RNA and mock affinity purifications, the RNA-protein complexes were eluted from streptavidin magnetic beads by boiling for 5 min in 0.1% SDS and treated with RNase A before being resolved by SDS-PAGE and Coomassie blue staining. The specific gel bands were excised, digested, and analyzed by mass spectrometry (Figure 2C). Mass spectrometry identifications from these two gel bands yielded a list of proteins specific to the 5′–3′ U-S1 RNA sample (Table 1). The five proteins were identified with potential role in RNA process by searching the protein database [23]: PHD finger protein 6 (PHF6) is reported to mediate transcriptional repression activity [24], Heterogeneous nuclear ribonucleoprotein G-like 1 (RBMXL1) Contains an RNA recognition motif and may be involved in pre-mRNA splicing [25], Heterogeneous nuclear ribonucleoprotein D (HNRNPD) binds with high affinity to RNA molecules and may be involved in translationally-coupled mRNA turnover [26], Small nuclear ribonucleoprotein-associated proteins B(SNRPB) is the core component of the spliceosomal U1, U2, U4, and U5 small nuclear ribonucleoproteins (snRNPs) and plays an important role in the splicing of cellular pre-mRNAs [27], and 40S ribosomal protein S8 (RPS8) is localizes in cytoplasmic mRNP granules containing untranslated mRNAs [28].

**Table 1:** Protein identification by mass spectrometry.

Description	Protein Score	Protein Mass (kDa)	Coverage (%)	Unique Peptide
PHF6	342.27	42.4	7.41	3
RBMXL1	463.26	42.2	19.98	6
HNRNPD	478.9	38.6	21.84	6
SNRPB	136.3	24.8	5.842	2
RPS8	427.5	24.5	27.88	5

Protein Score is the sum of the ion scores of all peptides that were identified. This number reflects the combined scores of all observed mass spectra that can be matched to amino acid sequences within that protein. A higher score indicates a more confident match. Coverage is the percentage of the protein sequence covered by identified peptides. The number of unique peptides that determines a protein group can be set in Proteome Discoverer. Unique Peptides are the peptide sequences that are unique to a protein group. These are the peptides that are common to the proteins of a protein group, and which do not occur in the proteins of any other group.

### 2.3. Involvement of the Newly Identified 5′–3′ UTR RBP RPS8 in DENV RNA Replication in Cells

**Figure 3:**
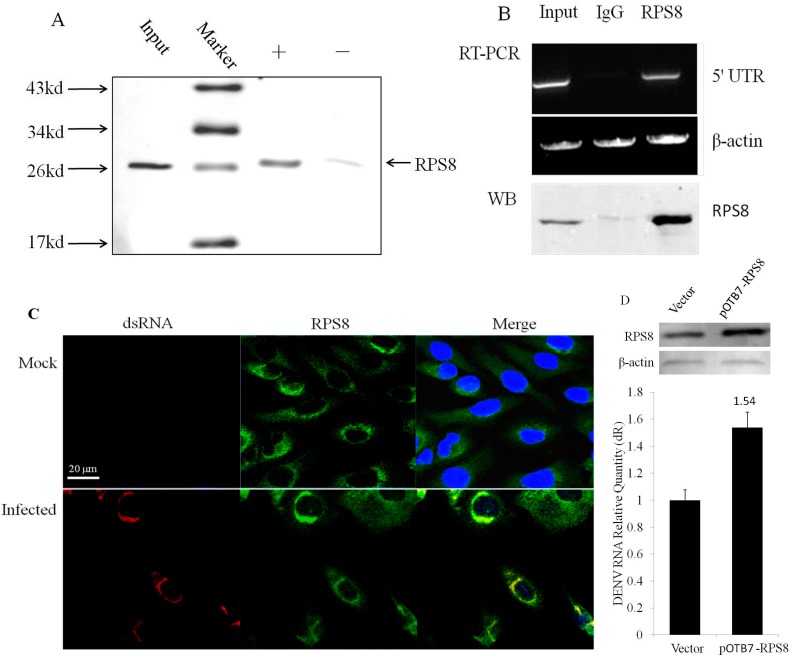

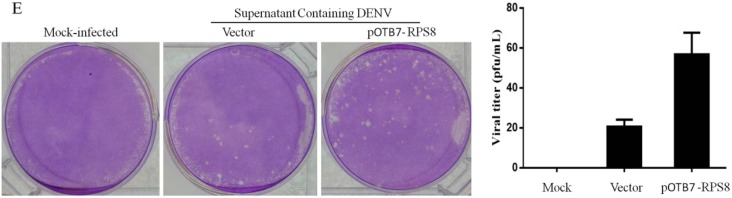
The newly identified 5′–3′ UTR RNP candidate RPS8 involved in DENV RNA replication. (**A**) Proteins in input whole cell extract or bound to washed streptavidin beads of 5′–3′ U-S1 or mock affinity purifications were used for immunoblot with the antibody against RPS8; (**B**) Viral RNA was co-immunoprecipitated from DENV-infected 293T cell extracts using an anti-RPS8 antibody. Rabbit IgG was used as a negative control. RNA was extracted from the immunoprecipitated complex or from an aliquot of the input lysate and subjected to RT-PCR using DENV 5′ UTR primers. The protein RPS8 immunoprecipitated by anti-RPS8 Ab was determined by Western blot; (**C**) RPS8 intracellular localization in mock-infected and DENV-2 infected A549 cells. Cells were fixed at 36 h post-infection and were stained with the nuclear (blue) stain DAPI, with anti-dsRNA (red) and anti-RPS8 (green) antibodies. The merge panel shows an overlay of the DAPI, dsRNA and RPS8 signals; scale bar = 20 μm; (**D**) RPS8 protein levels in control cells and RPS8 over-expression cells, and the corresponding DENV RNA level in cells was determined by RT-qPCR with primers of DENV 5′ UTR; (**E**) The plaque assay result of infectious viral particles yield in culture supernatant from DENV infected control cells and RPS8 over-expressing cells. The plaque assay data are expressed as the number of PFU per milliliter. One representative experiment of three is shown.

To confirm the newly identified 5′–3′ UTR binding protein which was enriched in the 5′–3′ U-S1 RNA purification, an immunoblotting approach was employed. As expected, an antibody against 40S ribosomal protein S8 (RPS8) recognized a protein of 24 kDa in whole cell extracts and in the viral RNP (vRNP) sample, but little in the mock sample purified in parallel (Figure 3A). To determine whether RPS8 associates with DENV RNA during virus infection, a viral RNA immunoprecipitation assay was performed using the anti-RPS8 antibody. As shown in Figure 3B, DENV RNA bound specifically to RPS8 as normal rabbit IgG precipitation controls run in parallel were negative. Furthermore, we examined the subcellular localization of RPS8 during DENV-2 infection using confocal laser scanning microscopy. In uninfected cells, there is no detectable dsRNA and only the RPS8 was observed. For the infected cells, RPS8 co-localized to the sites of dsRNA signal which indicates the replication sites of DENV (Figure 3C). These results suggest that RPS8 interacts with the DENV RNA during the course of DENV-2 infection in living mammalian cells. To ask whether RPS8 has the potential role in DENV-2 replication, we set up the over-expressing conditions for RPS8 in 293T cells followed by DENV-2 infection for 36 h and the levels of viral RNA in cells were analyzed by RT-qPCR with primers specific to the DENV 5′ UTR and infectious viral particle yield in culture supernatant was determined by the plaque assay. The results showed that the accumulation of DENV RNA (Figure 3D) and production of virus in supernatant (Figure 3E) were increased in RPS8 over-expressing cells. The RPS8 protein level in DENV infected cells was also assayed by Western blotting, and the result showed no significant difference between mock infected and infected cells (Figure S2). These results provide evidence that RPS8 is one of DENV RNA-binding proteins and positively participated in DENV replication.

## 3. Discussion

In this study, we devised a novel strategy to isolate endogenously-assembled viral RNA binding proteins (vRNPs) from crude mammalian cell extract by using S1 aptamer-based affinity purification. We expressed S1 aptamer-tagged DENV 5′–3′ UTR RNA in living mammalian cells, allowing endogenous assembly of the tagged RNA into vRNPs, and we purified tagged vRNPs from whole cell extract on Streptavidin magnetic beads. Proteins that were specifically associated with 5′–3′ U-S1 RNA were identified by mass spectrometry, and several newly identified protein partners potentially interact with DENV 5′–3′ UTR RNA. Further study showed that RPS8 interacted with DENV RNA in virus infected cells, and positively participated in DENV replication.

The expression of the aptamer-tagged RNAs in living mammalian cells may offer several advantages compared to solely *in vitro*-based methods. Here the utility of S1 aptamer as an RNA tag is demonstrated by isolating proteins that associate with DENV 5′–3′ UTR RNA. Firstly, the S1 aptamer is a short RNA sequence of 60 nt [7], which does not influence the secondary structure of tagged RNA. To generate the S1-tagged viral 5′–3′ UTR RNA in living mammalian cells, the pHH21 transcription system containing the human RNA polymerase I promoter and the mouse RNA polymerase I terminator was introduced and applied [29]; Secondly, endogenous transcription of RNA should be crucial for correctly assembling active RNPs whereas in vitro synthetic RNA may fold incorrectly due to the absence of auxiliary co-transcriptional factors in living cells. Moreover, the 5′ and 3′ UTRs of DENV are involved in long-range interactions and resulting in genome “circularization”, which plays key role in viral RNA replication and translation [30]. Using this strategy to transcribe DENV 5′–3′ UTR RNA in living cells is helpful to capture RBPs that are specifically binding to cyclized 5′ and 3′ UTR; Thirdly, the photoactivatable ribonucleoside induced crosslinking (PAR-CL) has been popularized recently, and the photoactivatable nucleotide 4-SU could be easily used to covalently couple proteins directly bound to RNA in living cells. This cross-link between protein and RNA is more effective than 254 nm UV light cross-linking and formaldehyde cross-linking and does not promote protein-protein crosslinking [31].

Recently, the most commonly used strategy in living cells is to insert one or more copies of an RNA-encoded aptamer, which is recognized by a small molecule (e.g., streptomycin) or a protein (e.g., MS2 coat protein or streptavidin) which is immobilized on chromatography matrix [4,32,33]. Tsai *et al.* [34] integrated MS2 in vivo biotin tagged RNA affinity purification (MS2-BioTRAP) with quantitative mass spectrometry based on stable isotope labeling with amino acid in cell culture (SILAC) technology. In this method, the target RNA was tagged with a specific RNA-stem loops structure which can recognized by bacteriophage protein MS2 [35,36]. Co-expression of HB-tagged MS2 and the stem-loops tagged target RNA in living cells allowed effective affinity purification of RNA-protein complexes, which was then identified and quantified by using SILAC-based mass spectrometry. The MS2-BioTRAP strategy can be used to isolate *in vivo*-assembled RNA-protein complexes. Novel RNA-binding proteins associated with a specific target RNA were successfully identified through these methods. However, the limit of these methods is that co-expressing a capture protein such as MS2 and stem-loop tagged target RNA is needed, thus MS2 might affect the RBPs that bind to the RNA sequence besides the stem-loop because the bacteriophage protein MS2 binds tightly to the stem-loop tagged RNA.

In this experiment, we developed an efficient and generalizable method to isolate viral RNA-associated RNPs or RBPs in mammalian cells and overcome current technical limitations for addressing RNA-protein interactions [37]. Furthermore, there is no length limitation to the bait RNA, as is the case in the peptide-nucleic acid-assisted RBP identification or electrophoretic mobility shift assay [38]. To avoid the loss of weakly-interacted partners during the wash procedures and stabilize the RNA-protein interactions, formaldehyde and 254 nm UV light cross-linking are commonly used to identify RNA-protein interactions in previously reported strategies. Recently, it was demonstrated that the 365 nm UV irradiation does not cross-link proteins to proteins and enhances direct RNA-protein interactions. Here, the cells transfected 5′–3′ UTR expression vector was cultured in the presence of photoactivatable nucleotide 4-thiouridine (4-SU) which was thus incorporated into nascent RNAs, and efficiently cross-linked by 365 nm UV light irradiation. Many host factors interacting with DENV 5′–3′ UTR was successfully identified. The RPS8 was further identified as the binding protein of DENV UTR by RIP assay and was functional during DENV replication. The RPS8 is ever identified in the insulin-like growth factor II mRNA-binding protein (IGF2BP or IMP) mRNP granules containing untranslated mRNAs in cytoplasm, which also include proteins such as HNRNPD (also identified in our experiment), IGF2BP1, DHX9, YBX1, ago-1, ago-2, HNRNPA2B1, HNRNPH1, HNRNPU and IGF2BP2 [28]. These proteins are functionally associated with viral RNA procession. Examples include IGF2BP1 interacts with Hepatitis C virus (HCV) 5′ UTR and 3′ UTR [39], the DHX9 could sense dsRNA and bind to the DENV 3′ UTR [40,41], ago-1 and ago-2 could promote DENV RNA replication [42], YB-1 and hnRNP A2/B1 also bind to the DENV 3′ UTR [43]. The detail mechanism of the RPS8 together with other IMP granule proteins participating in virus replication cycle needs to be further studied.

This strategy based on S1 aptamer and PAR-CL to isolate viral RBPs in living mammalian cells was reported for the first time and a scalable method in all steps was described, which can be performed in a single-run MS analysis with high-throughput studies in principle. With the improvement in sample preparation, purification process and data analysis, the high-resolution, quantitative MS may result in increasingly streamlined analysis of crude RNA-protein interactions in a single-step affinity purification at near-physiological conditions. Thus, this strategy has more potential for broad application in studying endogenously assembled RNA-protein complexes. This approach could be used to capture and identify the novel cellular factors associated with various viral RNAs.

## 4. Experimental Section

### 4.1. Plasmid Construction

The RPS8 protein expression plasmid pOTB7-RPS8 was purchased from the Biogot technology company. A description of the S1 aptamer expression plasmid and the control plasmid construction is given below. We cloned cDNAs derived from viral RNA downstream of the polymerase I promoter followed by terminator sequences. Briefly, the cDNAs were amplified by PCR with primers containing *Bsm*BI sites, digested with *Bsm*BI, and cloned into the pHH21 vector (kindly gift of Gabriele Neumann) containing the human RNA polymerase I promoter and mouse RNA polymerase I terminator (Figure 1B). By using splice overlap extension (SOE) PCR, an oligonucleotide containing S1 aptamer sequences and overlapping sequences from the DENV 5′ and 3′ UTR was used to fuse the 5′ UTR, 3′ UTR, and S1 aptamer sequences (5U-BsmB: 5′-CGTCTCCTATTAGTTGTTAGTCTACGTGGAC-3′ 3U-S1-BsmB: 5′-CGTCTCCGGGCATGGCCCGGCCCGCGACTATC-3′).

To create DNA templates for transcription of DENV 5′ and 3′ UTR in living cells, the full-length DENV-2 genome containing plasmid FL-D2 [44] was used as the template to amplify the 5′ UTR (1–172 nts) and 3′ UTR (10,242–10,724 nts) sequences. The S1 aptamer sequence was inserted into the 3′ end of 3′ UTR. All clones were checked by sequencing. The sequence and detail of construct have been supplied as supplementary document.

### 4.2. Cell Culture, Plasmid Transfection, Crosslinking, and Cell Lysis

A549, 293T and BHK-21 cells were maintained in DMEM (HyClone, Logan, UT, USA) supplemented with 10% FBS (HyClone), 10 U/mL penicillin, and 10 μg/mL streptomycin (Pen/Strep, Gibco, Grand Island, NY, USA) at 37 °C in a 5% CO_2_ atmosphere. The day before transfection, 293T cells were plated on 15 cm tissue culture dishes. About 50 μg plasmid per dish was transfected using X-tremeGENE 9 DNA Transfection Reagent (Roche, Basel, Switzerland) according to the manufacturer’s protocol. Six hours post-transfection, cells were maintained in 10% DMEM containing 100 µM/L 4-SU for additional 18 h. The photoactivatable nucleotide 4-thiouridine (4-SU) is taken up by cultured cells and then partially incorporated into nascent viral RNAs, and 365 nm UV light irradiation was applied to induce efficient crosslinking. Twenty-four hours post-transfection, cells were irradiated with UV light at 365 nm for 2 min, harvested, and lysed in 3 mL RIPA buffer under RNase-free and protease-free conditions. Total cell lysates were centrifuged for 10 min at 16,000× *g*, 4 °C. Supernatants were transferred to new tubes and protein was quantified by measuring absorbance at 280 nm.

### 4.3. DENV-2 Amplification and Infection

DENV-2 (strain Tr1751) was amplified in C6/36 cells as previously described [45]. For DENV-2 infection, the cells were incubated with the virus (MOI = 1) for 2 h at 37 °C with occasional rocking. After 2 h, the cells were rinsed, overlaid with complete medium, and incubated at indicated time points.

### 4.4. Reverse Transcript PCR (RT-PCR) and Relative Quantitative Real-Time PCR (RT-qPCR) Analysis

The DENV RNA isolated in the RNA immunoprecipitation assay was detected by RT-PCR according to the protocol. Briefly, 1 μg of RNA was first treated with DNase, and then converted to cDNA using PrimeScript RT Master Mix (TaKaRa, Shiga, Japan). The PCR reaction was performed with primers designed against the DENV 5′-UTR-F: 5′-AGTTGTTAGTCTACGTGGAC-3′ and 3′-UTR-R: 5′-AGAACCTGTTGATTCAACAGC-3′. 

The transcriptional level of pHH21-5′–3′ U-S1 in cells and the binding ability of 5′–3′ U-S1 RNA toward beads were analyzed by RT-qPCR with primers of the DENV 5′–3′ UTR segment (5′–3′ UTR-F, 5′-GCTGAAACGCGAGAGAAACC-3′; 5′–3′ UTR-R, 5′-TTTAACGTCCTTGGACGGGG-3′), which contain both the segment of 5′ and 3′-UTRs. The DENV RNA level in infected 293T cells was also analyzed by RT-qPCR (Primers: 5′ UTR-F, 5′-AGTTGTTAGTCTACGTGGAC-3′; 5′ UTR-R, 5′-CTACACGCGGTTTCTCTCGC-3′). The housekeeping gene β-actin (β-actin-F, 5′-TGACGGGGTCACCCACACTG-3′; β-actin-R, 5′-AAGCTGTAGCCGCGCTCGGT-3′) was used as an internal RT-qPCR control. Total RNA was isolated using RNAiso (TaKaRa) and synthesized into cDNA with random hexamers by using PrimeScript™ RT Master Mix (Perfect Real Time, TaKaRa) and was then amplified with above primers and SYBR Premix Ex Taq™ II (TaKaRa) for 40 cycles. Data were analyzed using Mx3005P System Software (Stratagene, La Jolla, CA, USA). Relative gene expression refers to the magnitude of the signal generated for the 5′–3′ UTR segment or 5′ UTR of DENV using the β-actin gene as the internal control. The data represent the mean of three independent experiments.

### 4.5. RNA Pull-down Assay

Twenty-four hours post-transfection, five dishes of cells were irradiated with UV light at 365 nm for 2 min, harvested, and lysed in 3 mL lysis buffer. DENV 5′–3′ U-S1 RNAs and crosslinked proteins were captured with Dynabeads^®^ MyOne™ Streptavidin C1 magnetic beads (Invitrogen, Waltham, MA, USA) as follows. In brief, one dish of cell lysate was incubated with 20 μL paramagnetic streptavidin beads (pre-treated according to manufacturer’s protocol) containing 100 μg yeast tRNA (Invitrogen) on a rotation wheel at 4 °C for 60 min. It was then washed four times with modified RIPA buffer (250 mM NaCl, 50 mM Hepes-HCl pH 7.5, 0.5% Sodium deoxycholate, 0.1% SDS, and 1% NP-40) at 4 °C. After washing, RNA conjoined to beads was isolated using RNAiso (TaKaRa) for subsequent RT-qPCR analysis, or RNA-protein complexes were dissociated from beads by boiling for 5 min in 0.1% SDS and then treated with bovine RNase A (TaKaRa) [46] for subsequent SDS-PAGE, Western blot, and mass spectrometry analysis.

### 4.6. SDS-PAGE and Western Blotting

The samples were analyzed by SDS-PAGE and transferred onto a nitrocellulose membrane. Membranes were incubated with blocking buffer (TBS containing 5% non-fat milk) for 1 h at room temperature and incubated with the primary antibody, a rabbit polyclonal anti-RPS8 (ProteinTech Group, Chicago, IL, USA) diluted 1:500 in blocking buffer overnight at 4 °C. The secondary antibody, IRDye 680RD Goat Anti-Rabbit Secondary Antibody (LI-COR Biosciences, Lincoln, NE, USA) diluted 1:8000, was incubated with the membranes at room temperature for 1 h. Protein detection was made using the infrared imaging system Odyssey (LI-COR Biosciences).

### 4.7. Mass Spectrometry

Protein gel bands were excised and in-gel-digested with trypsin, and the tryptic peptides were subjected to LC-MS/MS on an Orbitrap XL instrument (Thermo Fisher Scientific, Waltham, MA, USA) under standard conditions. All sequence and peptide fingerprint data was searched against the Swiss-Prot and NCBI non-redundant databases using MASCOTsoftware (Matrix Science, Boston, MA, USA).

### 4.8. Immunofluorescence Assay and Confocal Microscopy

A549 cells were seeded onto eight-well coverslips (LabTek, Waltham, MA, USA) and infected with DENV-2 for 24 h. Next the cells were fixed and permeabilized and then incubated with blocking buffer (PBS containing 3% bovine serum albumin (BSA)) for 1 h. Primary antibodies were diluted in blocking buffer (1:300 for anti-RPS8; 1:100 for anti-dsRNA, English & Scientific Consulting, Szirak, Hungary) and incubated with cells at 4 °C overnight. Cells were then washed and incubated for 1 h with fluorescently labelled Alexa Fluor 488 goat anti-Rabbit and Alexa Fluor 594 goat anti-mouse antibodies (Invitrogen). Coverslips were mounted in Prolong Gold antifade reagent containing DAPI (Invitrogen) and the cells were visualized under a laser confocal microscope LSM 510 (Zeiss, Oberkochen, Germany). The images were captured and analyzed by using ZEN software (Zeiss).

### 4.9. RNA Immunoprecipitation

The interaction of RPS8 with DENV viral RNA during infection was studied by using an RNA immunoprecipitation assay. In brief, the infected cells were harvested and cross-linked with 365 nm UV light. Cells were then disrupted in RIP assay buffer (50 mM Tris-HCl, pH 7.5, 1% Nonidet P-40, 0.5% sodium deoxycholate, 0.05% SDS, 1 mM EDTA and 150 mM NaCl, 0.2 mM PMSF, 1× protease inhibitors and 40 U RNase inhibitor), and the resulting lysates were pre-cleared with protein A-agarose (Santa Cruz, CA, USA). Immunoprecipitation was carried out using anti-RPS8 polyclonal antibody or normal rabbit IgG (as a negative control). Immune complexes were precipitated with protein A-agarose. Immunoprecipitated viral RNA was isolated using an RNeasy mini kit (Qiagen, Hilden, Germany). Reverse transcript PCR (RT-PCR) was conducted using primers of the DENV 5′-UTR.

### 4.10. Plaque Assay

293T cells, transfected with pOTB7-RPS8 or vector, were infected with DENV for 36 h and culture supernatant was collected. Then, virus yield in the culture supernatant was determined with BHK-21 cells. Briefly, BHK-21 cells were seeded in 6-well plates with 90% confluence. Serial dilutions of virus containing supernatants were added to the cells in a final volume of 1 mL/well. After 2 h of incubation at 37 °C and three time washes, DMEM containing 2% FBS and 1% low-melting-point agarose was added and plates incubated at 37 °C for five days. Plaques were counted after removal of the agarose plug and staining with crystal violet.

## Supplementary Materials

Supplementary materials can be found at http://www.mdpi.com/1422-0067/16/09/22456/s1.
