# Supplementary material for: Isolation of Endogenously Assembled RNA-Protein Complexes Using Affinity Purification Based on Streptavidin Aptamer S1
Source: Int J Mol Sci. 2015 Sep 16;16(9):22456–72. doi: 10.3390/ijms160922456 (PMC4613318; doi:10.3390/ijms160922456)
Supplement: Supplementary file 1 [file ijms-16-22456-s001.pdf]

Supplementary Information

Construction of plasmid pHH21-5-3U-S1

5′-3′ UTR-S1:

agttgttagtctacgtggaccgacaaagacagattctttgagggagctaagctcaacgtagttctaacagttttttaattagagagcagatctctgatgaa taaccaacgaaaaaaggcgagaaatacgcctttcaatatgctgaaacgcgagagaaaccgcgtgt**a**gGAATTCaaggcaaaactaacatga aacaaggctagaagtcaggtcggattaagccatagtacggaaaaaactatgctacctgtgagccccgtccaaggacgttaaaagaagtcaggccat tacaaatgccatagcttgagtaaactgtgcagcctgtagctccacctgagaaggtgtaaaaaatctgggaggccacaaaccatggaagctgtacgca tggcgtagtggactagcggttagaggagacccctcccttacaaatcgcagcaacaatgggggcccaaggtgagatgaagctgtagtctcactggaa ggactagaggttagaggagaccccccaaaacaaaaaacagcatattgacgctgggaaagaccagagatcctgctgtctcctcagcatcattccag gcacagaacgccagaaaatggaatggtgctgttgaatcaacaggttctGGATCCATGGGACCGACCAGAATCATGCAA GTGCGTAAGATAGTCGCGGGCCGGGCCATG

GAATTC highlighted with red color is the link of 5′ and 3′ UTR.

GGATCCATGGGACCGACCAGAATCATGCAAGTGCGTAAGATAGTCGCGGGCCGGGCCAT G highlighted with yellow color is the sequence of Bio Aptamer S1.

5′ UTR-F-T7

TAATACGACTCACTATAGGGagttgttagtctacgtggac

3′ UTR-R-S1:

CATGGCCCGGCCCGCGACTATCTTACGCACTTGCATGATTCTGGTCGGTCCCATGGATCCa gaacctgttgattcaacagc

The restriction sites of vector pHH21:

CGTCTCNTATTAGTAGAA....TTTTGCTCCCNGAGACG

GCAGAGNATAATCATCTT....AAAACGAGGGNCTCTGC

pHH21-5′–3′ U-S1

CGTCTCCTATTagttgttagtctacgtggaccgacaaagacagattctttgagggagctaagctcaacgtagttctaacagttttttaattagag agcagatctctgatgaataaccaacgaaaaaaggcgagaaatacgcctttcaatatgctgaaacgcgagagaaaccgcgtgt**a**gGAATTCaa ggcaaaactaacatgaaacaaggctagaagtcaggtcggattaagccatagtacggaaaaaactatgctacctgtgagccccgtccaaggacgtta aaagaagtcaggccattacaaatgccatagcttgagtaaactgtgcagcctgtagctccacctgagaaggtgtaaaaaatctgggaggccacaaac catggaagctgtacgcatggcgtagtggactagcggttagaggagacccctcccttacaaatcgcagcaacaatgggggcccaaggtgagatgaa gctgtagtctcactggaaggactagaggttagaggagaccccccaaaacaaaaaacagcatattgacgctgggaaagaccagagatcctgctgt ctcctcagcatcattccaggcacagaacgccagaaaatggaatggtgctgttgaatcaacaggttctGGATCCATGGGACCGACCA GAATCATGCAAGTGCGTAAGATAGTCGCGGGCCGGGCCATG**CCCGGAGACG**

The primers used to clone the 5′–3′ UTR-S1 into pHH21.

5′ UTR-F-BsmB:

5′-CGTCTCCTATTAGTTGTTAGTCTACGTGGAC-3′

3′ UTR-Bio-R-BsmB:

5′-CGTCTCCGGGCATGGCCCGGCCCGCGACTATC-3′

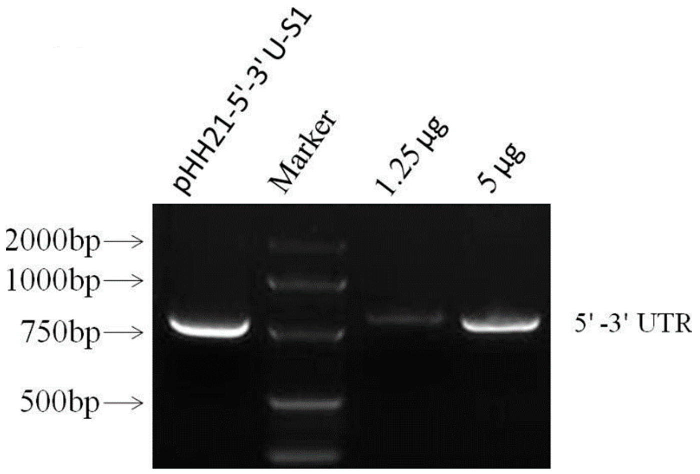

Figure S1. The RNA used in Figure 1D was reverse transcript to cDNA, and then was subjected to PCR using primers of 5′ UTR–F and 3′ UTR–R. The bands at around 750 bp proved that the DENV-UTR construct is transcribed as one intact RNA.

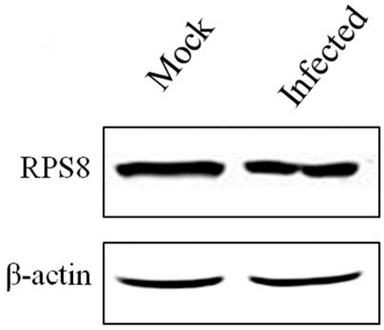

Figure S2. The protein level of RPS8 in DENV mock-infected and infected cells was assayed with western blotting using antibody against RPS8, and β-actin act as internal control. The result showed that there was no statistical difference between the two groups.

Tabel S1. The C_t value of RT-qPCR in the Figure 1D.

Group	β-Actin	5′–3′ UTR
1.25 µg	17.78	18.36
2.5 µg	17.95	17.96
5 µg	17.85	17.75
